# Assessment of Care Handoffs Among Hospitalist Physicians and 30-Day Mortality in Hospitalized Medicare Beneficiaries

**DOI:** 10.1001/jamanetworkopen.2021.3040

**Published:** 2021-03-24

**Authors:** Monica Farid, Yusuke Tsugawa, Anupam B. Jena

**Affiliations:** 1 Department of Health Care Policy, Harvard Medical School, Boston, Massachusetts; 2 Division of General Internal Medicine and Health Services Research, UCLA David Geffen School of Medicine, Los Angeles, California; 3 Department of Health Policy Management, UCLA Fielding School of Public Health, Los Angeles, California; 4 Department of Medicine, Massachusetts General Hospital, Boston; 5 National Bureau of Economic Research, Cambridge, Massachusetts

## Abstract

**Importance:**

Inpatients treated by hospitalist physicians, who often work contiguous days, experience handoffs at the end of a scheduled shift block. Evidence suggests that transitions of patient care, or handoffs, among physician trainees are associated with adverse patient outcomes. However, little is known about the association between handoffs and patient outcomes among attending physicians, even though similar concerns apply.

**Objective:**

To examine the association between inpatient handoffs of hospitalist physicians and patient mortality among hospitalized Medicare beneficiaries.

**Design, Setting, and Participants:**

This cross-sectional study analyzed a random sample of Medicare beneficiaries who were hospitalized with a general medical condition between January 1, 2011, and December 31, 2016, and treated by a hospitalist. The study compared outcomes of patients with low vs high probability of physician handoff based on date of patient admission relative to the admitting hospitalist’s last working day in a scheduled block, hypothesizing that otherwise similar patients admitted toward the end of a physician’s shift block would be more likely to be handed off to another physician compared with patients admitted earlier in the shift block. Data analysis was performed from July 1, 2018, to January 12, 2021.

**Exposure:**

High vs low probability of physician handoff.

**Main Outcomes and Measures:**

The main outcome was patient 30-day mortality rate.

**Results:**

A total of 1 074 000 patients (mean [SD] age, 75.9 [13.7] years; 57.4% female; 82.1% White) were studied. Multivariable regression models adjusted for beneficiary clinical and demographic characteristics and hospital fixed effects (a within-hospital analysis, effectively comparing patients treated at the same hospital). Among 597 288 hospitalizations, no overall difference in 30-day mortality was observed between patients admitted in the 2 days prior (days −1 and −2) to the treating hospitalist’s last working day (a high handoff probability) compared with days −6 and −7 (a low handoff probability) (adjusted rate, 10.6%; 95% CI, 10.5%-10.7% vs 10.6%; 95% CI, 10.5%-10.7%; adjusted difference, 0.0%; 95% CI, −0.2% to 0.1%). However, in an exploratory analysis, among patients with high illness severity, defined as those in the top quartile of estimated mortality, 30-day mortality was higher for those with high vs low likelihood of physician handoff (adjusted mortality, 27.8%; 95% CI, 27.6%-27.9% vs 26.8%; 95% CI, 26.6%-27.1%; absolute adjusted difference, 1.0%; 95% CI, 0.5%-1.4%).

**Conclusions and Relevance:**

In this national analysis of Medicare beneficiaries hospitalized with a general medical condition and treated by a hospitalist physician, physician handoff was not associated with increased mortality overall.

## Key Points

**Question:**

What is the association between inpatient physician handoff and mortality among hospitalized Medicare patients?

**Findings:**

In a national cross-sectional study of Medicare beneficiaries hospitalized with a general medical condition and treated by a hospitalist physician, physician handoff was not associated with increased mortality overall. In an exploratory analysis, among patients in the top quartile of estimated mortality, 30-day mortality was higher for patients with high vs low likelihood of handoff.

**Meaning:**

These findings suggest that among Medicare patients hospitalized with a general medical condition and treated by a hospitalist physician, inpatient physician handoffs appear safe overall but may be associated with slightly higher mortality among high-risk patients.

## Teaser

This cross-sectional study examines the association between inpatient handoffs among hospitalist physicians and patient mortality among hospitalized Medicare beneficiaries.

## Introduction

Transitions of patient care, or handoffs, have been associated with greater adverse events, preventable medical errors, and costs.^1,2,3,4,5,6,7^ Although handoffs occur in a number of medical settings, their impact has primarily been studied and associated with adverse events and errors in the trainee setting.^5,7^ Interventions to improve the safety and efficacy of handoffs,^8,9,10,11^ including a patient safety training and a structured handoff tool implemented at multiple centers,^12^ have similarly focused on trainees.

Handoffs are ubiquitous in the clinical practice that occurs once physicians complete their residency training, yet limited large-scale data exist on the association between physician handoffs and patient outcomes outside the trainee setting.^13^ The absence of empirical evidence on this association is particularly important because the structured emphasis on safe handoffs that routinely occurs in residency programs may infrequently carry forward once physicians enter independent practice, although the same safety considerations apply.

An important setting where handoffs commonly occur is inpatient care provided by hospitalist physicians.^14^ Hospitalists, usually general internists who specialize in hospital-based care, provide most inpatient general medical care in the US and typically work contiguous days in which handoff of patients to another physician occurs at the end of a scheduled block. Using data on Medicare beneficiaries hospitalized with a general medical condition and treated by a hospitalist physician during 2011 to 2016, we analyzed the association between physician handoffs and patient mortality by comparing outcomes of patients admitted at the beginning vs the end of a scheduled work block, at which point a handoff to another physician would usually occur. On the basis of prior studies^15,16,17,18^ and the way in which patients are typically assigned to hospitalist physicians, we hypothesized that patients treated by a given hospitalist at the beginning vs the end of a scheduled work block would otherwise be similar on both observable and unobservable characteristics that are associated with mortality, a quasi-experimental analysis.

## Methods

### Main Outcome, Study Sample, and Data Sources

The primary outcome was 30-day mortality, defined as death within 30 days of hospital discharge.

We identified all acute care hospitalizations of a 20% random sample of Medicare fee-for-service beneficiaries 65 years or older from January 1, 2011, to December 31, 2016, using the Medicare Inpatient Files linked by beneficiary identification to the 20% Medicare Carrier Files. We supplemented these data with annual Beneficiary Summary Files, which include demographics and chronic illness diagnoses. We focused on hospitalizations that involved a general medical rather than surgical condition (as defined by the presence of a medical diagnosis-related group [DRG] on admission) and in which care was provided by a hospitalist. Data analysis was performed from July 1, 2018, to January 12, 2021. The study was approved by the institutional review board at Harvard Medical School with a waiver of informed consent. All data were deidentified. This study followed the Strengthening the Reporting of Observational Studies in Epidemiology (STROBE) reporting guideline.

We first used established methods to assign an attending physician to each hospitalization based on the physician National Provider Identifier (NPI) in the Carrier File that accounted for the most Part B spending (evaluation and management [E&M] services, tests, and procedures) during that hospitalization.^15,17,18,19^ We then identified those hospitalizations in which the attending physician was a hospitalist, using a validated approach to define hospitalists: general internists with at least 5 E&M billings in a given year who filed at least 90% of their total E&M billings in an inpatient setting. This claims-based approach has been previously validated by calling physicians to confirm that they were hospitalists (sensitivity, 84%; specificity, 97%; positive predictive value, 89%).^20^ We restricted hospitalizations to those 2 weeks or less to reduce the possibility of multiple handoffs occurring during a given hospitalization because of the hospitalization spanning scheduled work blocks of multiple hospitalist physicians.

### Identification of Handoffs

A simple comparison of mortality rates between patients with and without handoffs would be confounded by the fact that patients with longer length of stay, often attributable to underlying disease severity that is associated with mortality, are more likely to experience handoffs. We addressed this issue by analyzing whether patient mortality varied according to date of admission relative to the assigned hospitalist’s last working day in a given shift block, hypothesizing that otherwise similar patients admitted toward the end of a physician’s shift block would be more likely to be handed off to another physician compared with patients admitted earlier in the shift block.

For each hospitalist in our data, we used the Carrier File to identify the last day of work in a possible shift block by isolating those inpatient E&M visits in which no inpatient billing was observed by that hospitalist in the Carrier File in the subsequent 7 consecutive days (in a sensitivity analysis, this threshold was lowered to 5 days) (eTable 4 in the Supplement). The calendar dates of these E&M visits were assumed to reflect the last day of a possible shift block for each hospitalist. For each hospitalization, we then defined a distance measure that was equal to the difference between the patient admission date and the last working date of the admitting hospitalist for their presumed shift block. For instance, if a patient was admitted May 16, 2014, and the admitting physician filed inpatient E&M claims on May 16 and 17 but not May 18 onward, the distance measure would be 2 days, reflective of when a patient was admitted relative to the hospitalist’s last working day in a given shift block (in this example, May 17). Given that the mean length of stay of patients in the study population was 3.6 days, a patient admitted on May 16 would, on average, be substantially more likely to be handed off to another physician than an otherwise similar patient admitted by that same hospitalist on May 10.

### Patient Covariates

Covariates included patient sex, age, race/ethnicity, indicators for 11 chronic conditions obtained from the Medicare Chronic Condition Data Warehouse, and admission DRG; indicator variables for day of week of admission, included to allow for the possibility that the day of week a patient was admitted may be associated with mortality^21^; indicator variables for calendar year to account for secular trends in patient mortality; and hospital fixed effects or hospital-specific indicator variables, included to account for time-invariant hospital factors that may be associated with patient mortality (our analysis therefore compared mortality rates of patients admitted at the beginning vs the end of hospitalists’ scheduled work blocks within the same hospital).

### Statistical Analysis

We first compared characteristics of patients who were admitted near the end of a hospitalist’s shift block (days −1 to −2, the 2 days before day 0, the last working day; high probability of handoff) vs early in the block (6 or 7 days earlier, days −6 to −7; low probability of handoff) to assess whether significant clinical differences existed in the types of patients who would be more likely to be handed off to another physician (ie, those patients admitted just before the shift block ended). In addition to comparing patient demographics and comorbidities, we also compared the probability distributions of admission DRGs between both groups (an approach used in prior studies^22,23^) to assess whether the reason for hospitalization differed between patients admitted toward the beginning vs the end of a hospitalist shift block.

We next estimated the association between a patient’s date of admission relative to a hospitalist’s last working day in a shift block and mortality. We estimated a multivariable linear regression model in which the dependent outcome was 30-day mortality and the key independent variables were a set of relative date indicators (ie, indicator variables for day −1, day −2, and so on until day −7), with other covariates described above. We plotted adjusted 30-day mortality rates according to the date a patient was admitted relative to a hospitalist’s last working day. We estimated linear models because of a failure of logistic regressions to converge with indicator variables for more than 500 DRGs and 4500 hospitals (in a sensitivity analysis, we estimated logistic models, excluding hospital fixed effects).

To facilitate interpretation in both the overall analysis and in subgroup analyses, we also estimated a hospitalization-level multivariable linear regression model in which the outcome variable was 30-day mortality and the key independent variable was a binary indicator for whether a patient was admitted in the 2 days before the admitting hospitalist’s last working day in a shift block (ie, days −1 to −2; high probability of handoff) vs the 6 or 7 days earlier (days −6 to −7; low probability of handoff), with other covariates described above and model interactions described below. The model was first estimated for the sample overall and then in 2 prespecified, exploratory subgroup analyses: (1) patients with high vs low illness severity, defined by being in the top vs bottom quartile of estimated mortality risk, as estimated by a multivariable linear probability model of 30-day mortality as a function of patient demographics, DRG, and comorbidity indicators, an analysis conducted to assess whether any adverse effect of handoffs was larger for patients with greater severity of illness; and (2) according to teaching hospital status (identified from the 2015 American Hospital Association Annual Survey), an analysis conducted to determine whether the association between physician handoffs and patient mortality varied according to whether trainees were involved in care and therefore in handoffs, as might occur in resident services of teaching hospitals. A formal test of interactions was performed in these subgroup analyses.

We conducted additional analyses. First, in addition to the confounders described above, any negative association between physician handoffs and patient mortality could also be explained by adverse effects of physician fatigue that occur at the end of working a continuous stretch of days, which would be a distinct mechanism (besides a handoff) by which the timing of a patient’s admission relative to the end of a hospitalist’s shift block could affect patient mortality. In a sensitivity analysis, we addressed this possibility by adjusting for the number of days a physician worked in the days before a given admission in the current shift block. This analysis therefore assessed the association between handoff and patient outcomes, holding fatigue, as measured by continuous days worked before an admission, constant. Second, although our baseline handoff analysis focused on patients who were hospitalized at the beginning vs the end of a hospitalist’s shift block, an alternative approach would be to simply examine patients who experienced handoffs and those who did not. We explored the characteristics of patients who underwent handoff vs those who did not to assess whether the former group was older and/or had more comorbidities than the latter group, as hypothesized. Handoffs were defined by hospital stays in which 2 hospitalist NPIs submitted E&M claims during the hospital stay as opposed to a single NPI. Third, our primary analysis identified a physician’s last working day in a shift block according to a gap of 7 days between that day and the next day in which an E&M claim was filed. In a sensitivity analysis, we restricted this gap to 5 days.

Analyses were performed in SAS (SAS Institute Inc) and Stata, version 14 (StataCorp, LLC) software. The 95% CI around reported estimates reflects 0.025 in each tail or a 2-tailed *P* ≤ .05.

## Results

A total of 1 074 000 patients (mean [SD] age, 75.9 [13.7] years; 57.4% female; 82.1% White) were studied. Overall, 597 288 hospitalizations were analyzed (see eTable 1 in the Supplement for sample construction); 366 287 hospitalizations occurred in the 2 days before the admitting hospitalist’s last working day in a shift block (days −1 to −2) and 231 001 occurred in the 6 or 7 days earlier (days −6 to −7). Patient characteristics were similar between these 2 groups, and any statistically significant differences were small in magnitude and not clinically meaningful (Table 1). For example, patients who were admitted in the 2 days before a hospitalist’s last working day had slightly fewer chronic conditions (mean, 6.7 vs 6.9 chronic conditions) compared with patients admitted 6 or 7 days earlier. The probability distributions of DRGs were similar between groups (eFigure in the Supplement), suggesting similar reasons for hospitalization. Although we could not directly observe handoffs using claims data, we found that hospital stays with a high probability of handoff (admission occurring 1-2 days before the physician’s end of shift) were 64% more likely to be associated with 2 hospitalist NPIs (rather than 1) compared with stays with a low probability of handoff.

**Table 1.** Characteristics of the Study Population^a^

Characteristic	Full sample (N = 1 074 000)	Patients with high likelihood of physician handoff (n = 366 287)^b^	Patients with low likelihood of physician handoff (n = 231 001)^b^
Age, mean (SD), y	75.9 (13.7)	75.7 (13.8)	75.9 (13.6)
Female sex	57.4	57.3	57.4
Male sex	42.6	42.7	42.6
White race^c^	82.1	82.0	82.0
Non-White^d^	17.9	18.0	18.0
Comorbidities, mean (SD)	6.8 (3.3)	6.7 (3.3)	6.9 (3.3)
Comorbidities			
Coronary artery disease	4.2	4.1	4.3
Alzheimer dementia	25.1	24.4	25.7
Atrial fibrillation	22.2	21.7	22.6
Chronic kidney disease	48.1	47.1	49.1
Chronic obstructive pulmonary disease	34.0	33.1	34.8
Diabetes	43.6	42.5	44.9
Congestive heart failure	44.6	44.1	45.2
Hyperlipidemia	53.9	53.4	54.5
Hypertension	61.6	61.5	61.8
Prior stroke or transient ischemic attack	82.5	81.9	82.9
Cancer	10.5	14.4	14.6

^a^ Data are presented as percentage of patients unless otherwise indicated.

^b^ Patients with a high likelihood of physician handoff were defined as those admitted in the 2 days before the attending hospitalist’s last working day in a shift block (days −1 to −2), and patients with a low likelihood were defined as those admitted in the 6 or 7 days before the attending hospitalist’s last working day in a shift block (days −6 to −7).

^c^ Included non-Hispanic White.

^d^ Included all other racial/ethnic groups.

There was no overall association between adjusted 30-day mortality and the timing of patient admission relative to the physician’s last working day (Figure). For example, among 218 818 patients admitted in the day before the treating hospitalist’s last working day (day −1), adjusted 30-day mortality was 10.9% (95% CI, 10.8%-11.0%) compared with 10.6% (95% CI, 10.4%-10.8%) among 122 998 patients admitted 7 days earlier (day −7), a statistically insignificant absolute adjusted difference of 0.3%. However, among patients with high illness severity, adjusted 30-day mortality increased with the probability of physician handoff and was highest for patients admitted the day before a hospitalist’s last working day in a shift block (Figure). For instance, the adjusted 30-day mortality rate for patients with high illness severity was 28.5% (95% CI, 28.1%-28.9%) for those admitted on day −1 and 27.2% (95% CI, 26.9%-27.6%) for those admitted on day −2 compared with 27.1% (95% CI, 26.6%-27.5%) for those admitted on day −6 and 26.8% (95% CI, 26.3%-27.3%) for patients admitted on day −7.

**Figure.**
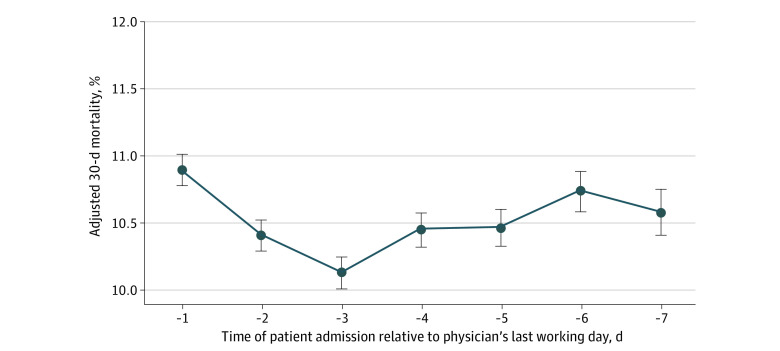
Adjusted 30-Day Mortality and Date of Patient Admission Relative to Last Working Day in a Physician’s Shift Block Figure plots adjusted patient 30-day mortality according to the date a patient was admitted relative to the last working day (day 0) in a physician’s shift block. A hospitalization-level multivariable linear regression model was estimated in which the dependent outcome was 30-day mortality, and the key independent variables were a set of relative date indicators (ie, indicator variables for day −1, day −2, and so on until day −7), with other covariates described in the Methods. Analyses were conducted for patients overall and stratified by severity of illness, defined by the bottom vs the top quartile of estimated 30-day mortality after hospitalization. Bars represent 95% CIs.

In an overall mortality comparison between patients with high vs low likelihood of physician handoff (Table 2), 30-day mortality was similar among patients admitted in the 2 days before the admitting hospitalist’s last working day in a shift block (days −1 to −2) vs the 6 or 7 days earlier (days −6 to −7) (adjusted mortality, 10.6%; 95% CI, 10.5%-10.7% vs 10.6%; 95% CI, 10.5%-10.7%; absolute adjusted difference, 0.0%; 95% CI, −0.2%-0.3%).

**Table 2.** Adjusted 30-Day Mortality According to Likelihood of Physician Handoff

Patient group	Likelihood of physician handoff^a^				Adjusted absolute difference, % (95% CI)
	Unadjusted, No./total No. (%)		Adjusted, % (95% CI)		
	High	Low	High	Low	
All patients (N = 597 288)	37 404/366 287 (10.2)	25 688/231 001 (11.1)	10.6 (10.5 to 10.7)	10.6 (10.5 to 10.7)	−0.03 (−0.18 to 0.12)
Low illness severity (n = 149 319)^b^	1265/93 915 (1.4)	895/55 404 (1.6)	1.4 (1.3 to 1.4)	1.5 (1.5 to 1.6)	−0.15 (−0.28 to −0.02)
High illness severity (n = 149 322)^b^	24 292/88 095 (27.6)	16 588/61 227 (27.1)	27.8 (27.6 to 27.9)	26.8 (26.6 to 27.1)	0.95 (0.51 to 1.40)

^a^ Patients with a high likelihood of physician handoff were defined as those admitted in the 2 days before the attending hospitalist’s last working day in a shift block (days −1 to −2), and patients with a low likelihood were defined as those admitted in the 6 or 7 days before the attending hospitalist’s last working day in a shift block (days −6 to −7). A hospitalization-level multivariable linear regression model was estimated in which the dependent outcome was 30-day mortality, and the key independent variable was a binary indicator for whether a patient was at high vs low likelihood of physician handoff, with other covariates described in the Methods. Handoff was interacted with illness severity (defined below) to allow for a formal test of interactions.

^b^ Patients with a low illness severity were defined as those in the bottom quartile of estimated 30-day mortality and patients with a high illness severity as those in the top quartile of estimated 30-day mortality.

In prespecified, exploratory subgroup analysis based on patients’ illness severity (Table 2), patients with a high illness severity and a high likelihood of physician handoff had greater adjusted 30-day mortality than those with a low likelihood of handoff. For example, among patients with high illness severity, unadjusted 30-day mortality was 27.6% (24 292 of 88 095 patients) among patients admitted on days −1 to −2 vs 27.1% (16 588 of 61 227 patients) among patients admitted on days −6 to −7 (adjusted mortality, 27.8%; 95% CI, 27.6%-27.9% vs 26.8%; 95% CI, 26.6%-27.1%; absolute adjusted difference, 1.0%; 95% CI, 0.5%-1.4%; relative adjusted difference, 3.7%). The likelihood of handoff was not associated with 30-day mortality for patients with low illness severity (adjusted mortality, 1.4%; 95% CI, 1.3%-1.4% vs 1.5%; 95% CI, 1.5%-1.6%; absolute adjusted difference, −0.15%; 95% CI, −0.28% to −0.02%).

The association between inpatient physician handoff and adjusted 30-day mortality was similar in teaching and nonteaching hospitals (Table 3). For example, among patients overall the absolute adjusted mortality difference between patients with high vs low likelihood of physician handoff was −0.1% (95% CI, −0.3% to 0.1%) in teaching hospitals and 0.0% (95% CI, −0.2% to 0.2%) in nonteaching hospitals (*P* = .71 in test of interactions). Among patients with high illness severity, those with high likelihood of physician handoff had greater mortality than those with low likelihood of handoff in nonteaching hospitals (absolute adjusted difference between admissions occurring on days −1 to −2 vs −6 to −7, 1.0%; 95% CI, 0.4%-1.6%; *P* = .003 in tests of interactions for patients overall and patients with high illness severity) and teaching hospitals (absolute adjusted difference, 0.8%; 95% CI, 0.15%-1.4%; *P* = .004 in tests of interactions for patients overall and patients with high illness severity).

**Table 3.** Adjusted 30-Day Mortality According to Likelihood of Physician Handoff for Teaching and Nonteaching Hospitals

Patient group	Likelihood of physician handoff^a^				Adjusted absolute difference, % (95% CI)
	Unadjusted, No./total No. (%)		Adjusted, % (95% CI)		
	High	Low	High	Low	
All patients (N = 597 288)					
Teaching	19 132/194 401 (9.8)	13 400/122 804 (10.9)	10.3 (10.2 to 10.4)	10.5 (10.4 to 10.6)	−0.11 (−0.32 to 0.11)
Nonteaching	18 272/171 886 (10.6)	12 288/108 197 (11.4)	10.9 (10.8 to 11.0)	10.9 (10.8 to 11.0)	−0.02 (−0.24 to 0.21)
Low illness severity (n = 149 319)^b^					
Teaching	641/52 025 (1.2)	461/30 477 (1.5)	1.3 (1.2 to 1.3)	1.4 (1.3 to 1.5)	−0.10 (−0.28 to 0.08)
Nonteaching	624/41 890 (1.5)	434/24 930 (1.7)	1.5 (1.5 to 1.6)	1.7 (1.6 to 1.8)	−0.15 (−0.35 to 0.05)
High illness severity (n = 149 322)^b^					
Teaching	12 547/45 446 (27.6)	8806/32 260 (27.3)	27.4 (27.2 to 27.7)	26.7 (26.3 to 27.0)	0.79 (0.15 to 1.43)
Nonteaching	11 745/42 649 (27.5)	7782/28 967 (26.9)	27.7 (27.4 to 27.9)	26.7 (26.3 to 27.1)	1.00 (0.36 to 1.64)

^a^ Patients with a high likelihood of physician handoff were defined as those admitted in the 2 days before the attending hospitalist’s last working day in a shift block (days −1 to −2), whereas patients with a low likelihood were defined as those admitted in the 6 or 7 days before the attending hospitalist’s last working day in a shift block (days −6 to −7). A hospitalization-level multivariable linear regression model was estimated in which the dependent outcome was 30-day mortality, and the key independent variable was a binary indicator for whether a patient was at high vs low likelihood of physician handoff, with other covariates described in the Methods. The handoff variable was interacted with indicator variables for teaching hospital status, allowing for a formal test of interactions to assess whether the association between mortality and likelihood of physician handoff varied by teaching status. Teaching hospitals were identified from the 2015 American Hospital Association Annual Survey.

^b^ Patients with low illness severity were defined as those in the bottom quartile of estimated 30-day mortality and patients with high illness severity as those in the top quartile of estimated 30-day mortality. Admissions at hospitals with insufficient information in the 2015 American Hospital Association data to determine teaching status were excluded from the regression, yielding a total of 121 124 admissions.

### Additional Findings

Similar findings were observed in logistic regression models that excluded hospital fixed effects (eTable 2 in the Supplement); after additional adjustment for the number of days a physician worked in the days before a given admission in the current shift block (eTable 3 in the Supplement), an analysis conducted to assess the association between handoff and patient outcomes, holding fatigue, as measured by continuous days worked before an admission, constant; and in analysis that identified a physician’s last working day in a shift block according to a gap of 5 days (as opposed to 7 days) between that day and the next day in which an E&M claim was filed (eTable 4 in the Supplement). In an analysis that compared characteristics of patients whose hospital stays involved E&M claims from 2 vs 1 hospitalist physician (ie, a direct proxy for handoff), patients who underwent a handoff were older, had higher prevalence of most comorbidities, and had substantially higher estimated mortality (11.6% vs 9.9% within 30 days of discharge) compared with patients who did not undergo handoff (eTable 5 in the Supplement), a finding supportive of the need to use a quasi-experimental empirical approach that does not directly compare patients who are handed off to those who are not.

## Discussion

In this cross-sectional study of a large national sample of Medicare beneficiaries hospitalized with a general medical condition and treated by a hospitalist physician during 2011 to 2016, physician handoff was not associated with differences in 30-day mortality among patients overall but, in an exploratory analysis, was associated with slightly greater mortality among patients with high illness severity. This study assessed the association between inpatient handoffs and mortality by comparing hospitalizations that occurred toward the end vs the beginning of a hospitalist physician’s scheduled work block, hypothesizing that both groups of patients, who would be expected to experience different probabilities of physician handoff, might otherwise be similar.

Although handoffs have been associated with greater adverse events, prior studies^14,24,25^ of handoffs have largely focused on the trainee setting, despite the fact that handoffs are common in the clinical practice that occurs once physicians complete their training. Hospitalists, for example, now provide most general medical care in inpatient settings and typically work contiguous days in which handoff of patients to another physician occurs at the end of a scheduled block. Despite the recognized importance of safe handoffs in hospital medicine,^14^ no research has used national data to evaluate whether handoffs in this setting are, in fact, associated with adverse events, such as higher mortality. In addition, research on the association between handoffs and quality of care in the nontrainee setting has been limited to specific diseases, single institutions, or outcomes, such as length of stay as opposed to mortality.^6,13^ Related research^26^ has also evaluated whether patient outcomes are worse for patients treated by hospitalists with lower general continuity in their work schedules, an important but distinct question from the association between inpatient handoffs and mortality.

The analytic approach to studying the association between physician handoff and patient mortality was based on the assumption that patients treated by a given hospitalist at the beginning vs the end of a work block would otherwise be similar except for the significantly greater likelihood of physician handoff at the end of a work block, a quasi-experimental analysis. If, instead, patients who were admitted toward the end of a work block were at higher risk of mortality because of observable or unobservable factors, the estimates of increased mortality would be confounded and biased upward. Patient characteristics were, however, clinically similar between those at high vs low likelihood of physician handoff. If anything, patients in the study who were admitted toward the end of a work block were slightly healthier based on comorbidities, which could be consistent with the preferential assignment of lower-risk patients to those hospitalists whose work block was just about to end. In this case, the estimated association between mortality and the likelihood of physician handoff would be biased downward not upward.

These findings, if causal, suggest that handoffs that commonly occur in inpatient medical settings are not associated with differences in mortality for patients overall but may be associated with slightly greater mortality among patients with high illness severity, although this latter finding was exploratory and requires validation. To date, however, most efforts to improve and monitor the safety of handoffs have focused on trainees. For example, although the Accreditation Council for Graduate Medical Education requires formal training in handoffs and monitoring of handoff quality,^27^ the quality of handoff strategies is not systematically assessed in existing measures of hospital quality. Moreover, a number of interventions have been tried among trainees to target vulnerabilities in the handoff process, one example being I-PASS (illness severity, patient summary, action list, situation awareness and contingency plans, and synthesis by receiver), which has been associated with significant reductions in medical errors and adverse events. These findings suggest the potential utility of evaluating these tools and strategies more broadly in the hospital medicine setting, at least for patients with high illness severity, because the risks associated with handoff may not be confined to trainees. This implication is supported by the similar handoff-associated mortality differences observed among patients with high illness severity in teaching and nonteaching hospitals, an important distinction because transitions of care that involve resident physicians should not explain findings in nonteaching hospitals.

The findings of this study also suggest the potential importance of patient triage as a way to mitigate the risks of physician handoff. For example, because of the observed association between likelihood of handoff and mortality only among patients with high illness severity, it is possible that systematic triage of these patients to hospitalists who are at the beginning vs the end of a scheduled work block may be one way to reduce mortality associated with handoffs. This recommendation must be tempered by the fact that our finding that handoffs were associated with greater mortality was confined to high-risk patients and therefore needs validation in other data and with other methods.

### Limitations

This study has limitations. First, it was observational, and although patients with high vs low likelihood of handoff were similar on a range of characteristics, including reason for admission, residual confounding may be present. Second, aside from confounding, the effect of physician handoffs on patient outcomes is difficult to empirically distinguish from 2 other mechanisms: (1) potential adverse effects of physician fatigue, owing to working a continuous stretch of days in the hospital, and (2) improvements in care because of a second physician (ie, a second set of eyes) being involved in the care of patients early in their hospitalization course.^28^ Third, this study focused on mortality rather than other medical errors or adverse events. Fourth, the study evaluated an important but common set of handoffs that occur in the hospital setting: the handoff at the end of a hospitalist service block. Handoffs occur in other specialties and within all inpatient specialties, and daily handoffs occur between day and night practitioners. Recent evidence from primary care that focuses on the potential effects of continuity of care rather than discontinuities in care suggests that continuity may be associated with improved primary care outcomes.^29^ Fifth, data on the quality of handoff processes in individual hospitals were lacking, making it impossible to identify what specific processes of care within hospitals could be associated with better handoff outcomes among patients with high illness severity. Relatedly, the frequency and duration of hospitalist work blocks, as well as protocols around handoffs, likely vary significantly across hospitals, and this analysis measured the association between handoffs and mortality across hospitals, even though substantial hospital-level heterogeneity may exist.^30^ Sixth, it was not possible to directly observe handoffs using claims data; therefore, we used proxy measures of handoffs (eg, estimates of the probability of handoff using NPI billing to identify the number of contiguous days of care).

## Conclusions

In this cross-sectional study, in a large national sample of Medicare beneficiaries hospitalized with a general medical condition and treated by a hospitalist physician, physician handoff was not associated with differences in 30-day mortality among patients overall. However, in an exploratory analysis, physician handoff was associated with slightly greater mortality among patients with high severity of illness. Validation of this approach using detailed scheduling data would be useful.

## Notes

Supplement. **eTable 1.** Sample Construction **eTable 2.** Adjusted 30-Day Mortality According to Likelihood of Physician Handoff, Sensitivity Analysis Using Logistic Model **eTable 3.** Adjusted 30-Day Mortality According to Likelihood of Physician Handoff, Adjusting for Shift Length Before Admission **eTable 4.** Adjusted 30-Day Mortality According to Likelihood of Physician Handoff, Sensitivity Analysis Using a 5-Day Gap to Identify a Hospitalist’s Last Day in a Shift Block **eTable 5.** Comparison of Patient Characteristics Between Patients With 2 vs 1 Hospitalist Physicians Involved in Their Stay **eFigure.** Diagnosis Related Group (DRG) Cumulative Distribution, According to Likelihood of Physician Handoff
