# Supplementary material for: Assessment of Care Handoffs Among Hospitalist Physicians and 30-Day Mortality in Hospitalized Medicare Beneficiaries
Source: JAMA Netw Open. 2021 Mar 24;4(3):e213040. doi: 10.1001/jamanetworkopen.2021.3040 (PMC7991971; doi:10.1001/jamanetworkopen.2021.3040)
Supplement: Supplement. — eTable 1. Sample Construction eTable 2. Adjusted 30-Day Mortality According to Likelihood of Physician Handoff, Sensitivity Analysis Using Logistic Model eTable 3. Adjusted 30-Day Mortality According to Likelihood of Physician Handoff, Adjusting for Shift Length Before Admission eTable 4. Adjusted 30-Day Mortality According to Likelihood of Physician Handoff, Sensitivity Analysis Using a 5-Day Gap to Identify a Hospitalist’s Last Day in a Shift Block eTable 5. Comparison of Patient Characteristics Between Patients With 2 vs 1 Hospitalist Physicians Involved in Their Stay eFigure. Diagnosis Related Group (DRG) Cumulative Distribution, According to Likelihood of Physician Handoff [file jamanetwopen-e213040-s001.pdf]

Supplementary Online Content

Farid M, Tsugawa Y, Jena AB. Assessment of care handoffs among hospitalist physicians and 30-day mortality in hospitalized Medicare beneficiaries. *JAMA Netw Open*. 2021;4(3):e213040. doi:10.1001/jamanetworkopen.2021.3040

eTable 1. Sample Construction
eTable 2. Adjusted 30-Day Mortality According to Likelihood of Physician Handoff, Sensitivity Analysis Using Logistic Model
eTable 3. Adjusted 30-Day Mortality According to Likelihood of Physician Handoff, Adjusting for Shift Length Before Admission
eTable 4. Adjusted 30-Day Mortality According to Likelihood of Physician Handoff, Sensitivity Analysis Using a 5-Day Gap to Identify a Hospitalist's Last Day in a Shift Block
eTable 5. Comparison of Patient Characteristics Between Patients With 2 vs 1 Hospitalist Physicians Involved in Their Stay
eFigure. Diagnosis Related Group (DRG) Cumulative Distribution, According to Likelihood of Physician Handoff

This supplementary material has been provided by the authors to give readers additional information about their work.

© 2021 Farid M et al. *JAMA Network Open.*

eTable 1: Sample construction

All hospital stays between 2011 and 2016.	66,816,946
All stays between 2011 and 2016 that can be linked to 20% carrier file, stays that are at acute care hospital. Stays linked to a hospitalist NPI and 2 weeks or less.	4,583,742
Admitting NPI has at least one 7 day gap in billing during the year.	3,282,979
Limiting to stays with a general medical condition. (Dropping stays with a general surgical condition)	2,684,819
Keep only stays that are one or two days from end of shift or six or seven days from end of shift.	597,288

© 2021 Farid M et al. *JAMA Network Open.*

eTable 2: Adjusted 30-day mortality according to likelihood of physician handoff, sensitivity analysis using logistic model

30 Day Mortality	Adjusted, % (95% CI)		Adjusted
	Likelihood of physician handoff [a]		Odds ratio, % (95% CI)
	High	Low	
All patients	10.4%	10.5%	0.99
(N =597,288)	(10.3% to 10.5%)	(10.4% to 10.6%)	(0.98 to 1.01)
Low severity of illness [b]	1.4%	1.6%	0.89
(N = 149,319)	(1.3% to 1.5%)	(1.5% to 1.7%)	(0.82 to 0.97)
High severity of illness [b]	27.3%	26.4%	1.05
(N = 149,322)	(26.9% to 27.6%)	(26.1% to 26.8%)	(1.02 to 1.07)

Notes: [a] Patients with a high likelihood of physician handoff were defined as those admitted in the 2 days prior to the attending hospitalist's last working day in a shift block (days -1/-2), while patients with a low likelihood were defined as those admitted in the 6 or 7 days prior (days -6/-7). A hospitalization-level multivariable logistic regression was estimated in which the dependent outcome was 30-day mortality and the key independent variable was a binary indicator for whether a patient was at high vs. low likelihood of physician handoff, with other covariates described in the Methods. Hospital fixed effects were not included in the model due to a failure of the model to converge. Handoff was interacted with illness severity (defined below) to allow for a formal test of interactions. [b] Low-severity patients were defined as those in the bottom quartile of predicted 30-day mortality and high-severity patients as those in the top quartile of predicted 30-day mortality.

© 2021 Farid M et al. *JAMA Network Open.*

eTable 3: Adjusted 30-day mortality according to likelihood of physician handoff, adjusting for shift length prior to admission

30 Day Mortality	Adjusted, % (95% CI) Likelihood of physician handoff [a]		Adjusted difference, % (95% CI)
	High	Low	
All patients	10.5%	10.6%	-0.05%
(N =597,288)	(10.5% to 10.6%)	(10.5% to 10.7%)	(-0.20% to 0.10%)
Low severity of illness [b]	1.4%	1.5%	-0.14%
(N = 149,319)	(1.4% to 1.4%)	(1.5% to 1.6%)	(-0.27% to -0.01%)
High severity of illness [b]	27.8%	26.8%	0.92%
(N = 149,322)	(27.6% to 27.9%)	(26.6% to 27.1%)	(0.47% to 1.37%)

Notes: Table reports analysis of whether the association between physician handoff and patient 30-day mortality may be affected by fatigue occurring towards the end of a physician's scheduled shift block as opposed to transition-of-care lapses that also occur at the end of a shift block. The analysis addresses this issue by adjusting for the number of days a physician worked in the days prior to a given admission in the current shift block, thereby comparing outcomes of patients who are handed off versus not handed off among physicians who have been working the same number of days prior to handoff. This covariate adjustment should account for any effect of physician fatigue on patient outcomes by studying the relationship between handoff and patient outcomes holding fatigue, as measured by continuous days worked prior to an admission, constant. [a] Patients with a high likelihood of physician handoff were defined as those admitted in the 2 days prior to the attending hospitalist's last working day in a shift block (days -1/-2), while patients with a low likelihood were defined as those admitted in the 6 or 7 days prior (days -6/-7). A hospitalization-level multivariable linear regression was estimated in which the dependent outcome was 30-day mortality and the key independent variable was a binary indicator for whether a patient was at high vs. low likelihood of physician handoff, with other covariates described in the Methods. Handoff was interacted with illness severity (defined below) to allow for a formal test of interactions. [b] Low-severity patients were defined as those in the bottom quartile of predicted 30-day mortality and high-severity patients as those in the top quartile of predicted 30-day mortality.

© 2021 Farid M et al. *JAMA Network Open.*

eTable 4: Adjusted 30-day mortality according to likelihood of physician handoff, sensitivity analysis using a 5-day gap to identify a hospitalist's last day in a shift block

30 Day Mortality	Unadjusted, % (No.)		Adjusted, % (95% CI)		Adjusted difference, % (95% CI)
	Likelihood of physician handoff [a]		Likelihood of physician handoff [a]		
	High	Low	High	Low	
All patients	10.3%	11.1%	10.6% (10.5% to 10.6%)	10.5% (10.5% to 10.6%)	0.06% (-0.08% to 0.19%)
Low severity of illness [b]	1.3%	1.7%	1.4% (1.3% to 1.4%)	1.6% (1.5% to 1.7%)	-0.21% (-0.33% to 0.09%)
High severity of illness [b]	27.7%	26.9%	27.9% (27.7% to 28.0%)	26.7% (26.5% to 26.9%)	1.14% (0.74% to 1.54%)

Notes: Table reports relationship between likelihood of physician handoff and adjusted 30-day patient mortality in a sensitivity analysis that identified a hospitalist physician's last working day in a shift block according to a gap of 5 days (as opposed to 7 days in the primary analysis) between inpatient evaluation and management claims filed in the inpatient setting. The relationship between likelihood of physician handoff and adjusted 30-day patient mortality was similar to the primary analysis. [a] Patients with a high likelihood of physician handoff were defined as those admitted in the 2 days prior to the attending hospitalist's last working day in a shift block (days -1/-2), while patients with a low likelihood were defined as those admitted in the 6 or 7 days prior (days -6/-7). A hospitalization-level multivariable linear regression model was estimated in which the dependent outcome was 30-day mortality and the key independent variable was a binary indicator for whether a patient was at high vs. low likelihood of physician handoff, with other covariates described in the Methods. Handoff was interacted with severity of illness (defined below) to allow for a formal test of interactions. [b] Low-severity patients were defined as those in the bottom quartile of predicted 30-day mortality and high-severity patients as those in the top quartile of predicted 30-day mortality.

© 2021 Farid M et al. *JAMA Network Open.*

eTable 5: Comparison of patient characteristics between patients with 2 vs 1 hospitalist physicians involved in their stay

	Handoff	No Handoff
	**(N = 388,540)**	**(N = 685,243)**
Age, mean (SD), y	76.2	75.6
Female, No. (%)	57.6%	57.3%
White race, No. (%)	82.4%	82.0%
Comorbidities, mean (SD)	6.9	6.7
**Comorbidities,** No. **(%)**		
Coronary artery disease	4.20%	4.1%
Alzheimer's dementia	26.3%	24.5%
Atrial Fibrillation	23.3%	21.6%
Chronic Kidney Disease	50.2%	46.9%
Chronic obstructive pulmonary disease	35.1%	33.4%
Diabetes	45.3%	44.2%
Congestive Heart Failure	45.4%	42.6%
Hyperlipidemia	61.8%	61.5%
Hypertension	83.0%	82.1%
Prior Stroke/Transient Ischemic Attack	54.5%	53.6%
Cancer	14.9%	14.4%
Predicted Mortality	11.6%	9.9%

Notes: Although our baseline handoff analysis focused on patients who were hospitalized at the beginning versus the end of a hospitalist's shift block, an alternative approach would be to simply examine patients who received handoffs and those who do not. We explored the characteristics of patients who underwent handoff versus those who did not to assess whether the former group was older and/or had more comorbidities than the latter group, as hypothesized. Handoffs were defined by hospital stays in which two hospitalist NPIs submitted E&M claims during the hospital stay as opposed to a single hospitalist NPI.

© 2021 Farid M et al. *JAMA Network Open.*

eFigure 1: Diagnosis related group (DRG) cumulative distribution, according to likelihood of physician handoff

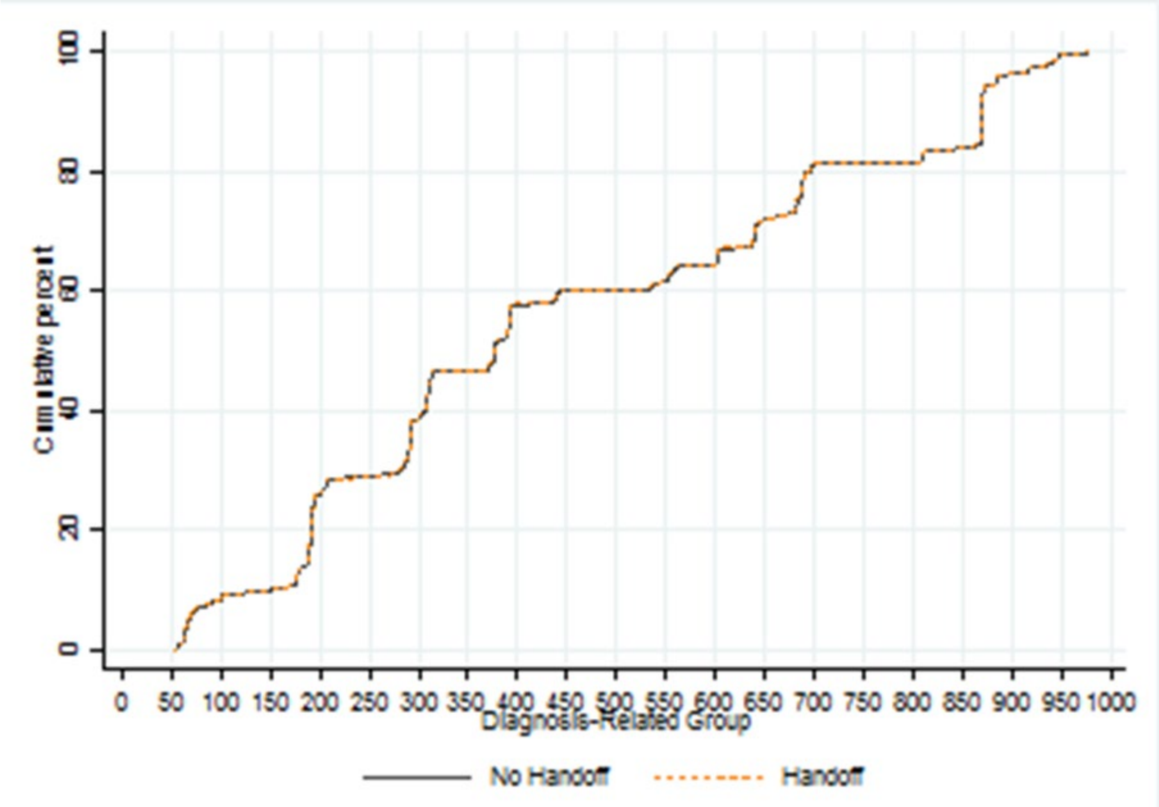

Notes: Figure shows cumulative distributions of the admitting Diagnosis-Related Groups (DRG) for admissions in the study sample, separated by the likelihood of physician handoff (patients admitted on days -1/-2 are at high probability of physician handoff, orange dashed line; patients admitted on days -6/-7 were at low probability of physician handoff, black line). Even though DRG numbers are categorical values representing separate diagnoses, we graphed the cumulative distribution on a continuous scale to visualize the case-mix of admissions across hundreds of DRGs. Therefore, the overlap between the two groups' distributions can reveal any subtle differences in case-mix across these many diagnoses. Distributions were not statistically significantly different in a 2-sample Kolmogorov-Smirnov test for equality of distribution functions ($p = 1.00$).

© 2021 Farid M et al. *JAMA Network Open.*
